# Supplementary material for: An Integrative Migraine Polygenic Risk Score Is Associated with Age at Onset But Not Chronification
Source: J Clin Med. 2024 Oct 29;13(21):6483. doi: 10.3390/jcm13216483 (PMC11547092; doi:10.3390/jcm13216483)

Supplemental Item **Page**

Table S1 DodoNA migraine-toolkit data elements 2

Fig S1 Evaluation of the proportional hazards assumption in models for the association of migraine risk scores and age at onset of migraine attacks or chronification of migraine 5

Table S1: DodoNA migraine-toolkit data elements[a]

Data Element	Recorded As
**Medical History**	
Encounter date	Date
Age	Years
Body mass index (BMI)	BMI
Sex	M/F
Education	Years
Family history: epilepsy, hypertension, migraine, neurological disease, Parkinson's disease, stroke	Presence / absence of each in enumerated first-degree relatives
**History of Migraine Attacks**	
Age at onset (AAO)	Years
Frequency	<1/mo, 1-3/mo, 1/wk, 2-3/wk, >3/wk, daily, constant
Estimated lifetime total	<5, 5-10, 11-50, 51-100, >100
Location	Unilateral/bilateral
Details	Primary headache; Left, right, alternating (unilateral); frontal, orbital, parietal, temporal, occipital, holocephalic (bilateral)
Quality	None, throbbing, pressure, vise-like, stabbing, dull aching, imploding, exploding
Severity	0-10
Associated symptoms	None, nausea, vomiting, sensitivity to light, noise, smell; change in vision, dizziness, fatigue, face-flushing, red-eye, ptosis, runny nose/eyes, allodynia, ringing in ears, neck stiffness
Average duration	<5 min, 5-15 min, 15-60 min, 1-4 hr, 4-24 hr, 1-3 days, >3 days, constant
Timing	Continuous, any time, waking, morning, afternoon, evening, night, during sleep, weekends, peri(menstrual)
Triggers	None, weather, altered sleep, stress, skipped meals, smells, sounds, menstruation, alcoholic beverages, foods, caffeine, chocolate, Nutrasweet, MSG, cured meat, onion, aged cheese, banana, nuts, other
Time to peak intensity	<5 min, 5-15 min, 15-60 min, 1-4 hr, >4 hr
Aura symptoms	None, visual, dysarthria, aphasia, sensory, motor
Aura duration	<5 min, 5-60 min, 1-3 hr, 3-24 hr, >24 hr
Influences	None, abortive medication, cold compresses, heat, massage, relaxation, sleep, other
Exacerbating measures	None, routine physical activity, exercise, coughing, sneezing, straining, bending, change in position, other
Temporal course	Progressing, improving, static, resolved
Emergency department (ED) visits for headache	Ever, number in past year (0 – 12+)
Hospitalizations for headache	Ever, number in past year (0 – 12+)
**Clinical Impression** (diagnosis using International Classification of Headache Disorders 3rd Ed. criteria)	
Headache disorder	Present / absent
Primary headache disorder	Present / absent
Migraine	Present / absent
Tension-type headache	Present / absent

Cluster headache / other trigeminal autonomic cephalalgias	Present / absent
Other primary headache	Present / absent
Migraine Subtype	
Migraine with aura (MA)	Present / absent
Migraine without aura (MOA)	Present / absent
Childhood periodic syndromes that are common precursors of migraine	Present / absent
Retinal migraine	Present / absent
Probable migraine	Present / absent
If migraine with aura -	
Typical aura with migraine headache	Present / absent
Typical aura with non-migraine headache	Present / absent
Typical aura without headache	Present / absent
Familial hemiplegic migraine	Present / absent
Sporadic hemiplegic migraine	Present / absent
Basilar-type migraine	Present / absent
Migraine with brainstem aura	Present / absent
Chronic migraine	Present / absent, first occurrence (year)
Complications of migraine	
Migrainous infarction	Present / absent, first occurrence (year)
Status migrainosus	Present / absent, first occurrence (year)
Persistent aura without infarction	Present / absent, first occurrence (year)
Migraine-triggered seizures	Present / absent, first occurrence (year)
Secondary headache disorder	Presence / absence
Head and/or neck trauma	Presence / absence
Cranial and/or cervical vascular disorder	Presence / absence
Non-vascular intracranial disorder	Presence / absence
High CSF pressure	Presence / absence
Low CSF pressure	Presence / absence
Substance or its withdrawal	Presence / absence
Headache induced by acute substance use / exposure	Presence / absence
Medication overuse headache	Presence / absence
Infection	Presence / absence
Disorder of homeostasis	Presence / absence
Disorder of cranium, neck, other facial/cranial structures	Presence / absence
Psychiatric disorder	Presence / absence
Cranial neuralgias	Presence / absence
Trigeminal neuralgia	Presence / absence
Tolosa Hunt syndrome	Presence / absence
Supra orbital neuralgia	Presence / absence
Occipital neuralgia	Presence / absence
Herpes zoster	Presence / absence
Recurrent painful ophthalmoplegic neuropathy	Presence / absence
Other	Presence / absence
Co-morbidities: anxiety, asthma, bipolar disorder, depression, epilepsy, fibromyalgia, hypertension, insomnia, obesity, post-traumatic stress disorder, restless leg syndrome, sleep apnea, stroke, vertigo	Presence / absence of each
Abortives	Type / name

Number pills taken per month	Presence / absence
2-hr response	Yes / no / unknown
2-hr pain free	Yes / no / unknown
24-hr pain free	Yes / no / unknown
Loss of responsiveness	Yes / no / unknown
Rescue med	Yes / no / unknown
Preventives	Type / name
Active	Yes / no / unknown
Improved headaches	Yes / no / unknown
Tolerated	Yes / no / unknown
Complementary and alternative therapies	Type / name
Active	Yes / no / unknown
Improved headaches	Yes / no / unknown
Tolerated	Yes / no / unknown
Interventional therapies	Type / name
Active	Yes / no / unknown
Improved headaches	Yes / no / unknown
Tolerated	Yes / no / unknown
**Objective Test Scores**	
Migraine Disability Assessment (MIDAS)	Scores on questions 1-5, total score, A, B
Migraine-Specific Quality of Life (MSQ)	Role-function restrictive (RR), role-function-preventive (RP), emotional function (EF) scores, total score
Center for Epidemiologic Studies Depression Scale (CES-D)	Score
Insomnia Severity Index (ISI)	Score
Generalized Anxiety Disorder 7-item (GAD7)	Score

[a]Sex, education, family history and AAO were collected only at study enrollment. All other data elements were collected at study enrollment and at each annual follow-up.

Fig S1: Evaluation of the proportional hazards assumption in models for the association of migraine risk scores and age at onset of migraine attacks or chronification of migraine

The goodness of fit of the Cox proportional hazard models was assessed visually by plotting the estimated cumulative hazard function for the Cox-Snell residuals relative to the residuals themselves. The close alignment suggests that the proportional hazards assumption is not violated except at relatively long survival times. Plots evaluate the assumption for the association of the PRS with the AAO of migraine attacks in DodoNA patients (**A−D**), age at first ICD code for migraine in GHI patients (**I−L**), age at chronification in all DodoNA patients (**Q−R**), age at chronification in the DodoNA migraine cohort (**S**), and years from AAO to chronification in the DodoNA migraine cohort (**T**). When estimates of baseline hazard differed by sex (**Fig 3-5**), models were developed separately for males and females. Panels **A-B**, **E-F**, **I-J**, **M-N**, and **Q** present results for models using males; panels **C-D**, **G-H**, **K-L**, **O-P**, and **R** present results for models using females. For each model, a global test of the proportional hazards assumption based on the Schoenfeld residuals is also reported. Analysis time in panels **A**, **C**, **E**, **G**, **I**, **K**, **M**, **O**, **Q**, **R**, and **S** is age. Analysis time in panels **B**, **D**, **F**, **H**, **J**, **L**, **N** and **P** is age-quartile. Compare **A** and **C** to **Fig 3C**: **B** and **D** to **Fig 3D**; **I** and **K** to **Fig 4B**; and **J** and **L** to **Fig 4C**, respectively.

Plots of ln(analysis time) versus –ln{-ln(survival)} are shown for the AAO of migraine attacks in DodoNA patients (**E-H**), age at first ICD code for migraine in GHI patients (**M-P**), and years from AAO to chronification for patients in the DodoNA migraine cohort (**V**) for groups defined by risk score tertiles. Panel **U** shows the survival free of chronification in DodoNA cases relative to disease duration. The proportional hazards assumption is not violated when the curves are parallel. This assumption is generally satisfied for analyses where the results of pairwise log-rank tests revealed significant differences between groups defined by risk-score tertiles (**Table 3**, **Fig 4-5**). Compare panels **E** and **G** to **Fig 3E** and **3G**; **F** and **H** to **Fig 3F** and **3H**; **M** and **O** to **Fig 4D** and **4F**; **N** and **P** to **Fig 4E** and **4G**; and **U** to **V**, respectively.

Supplemental Figure S1, part 1

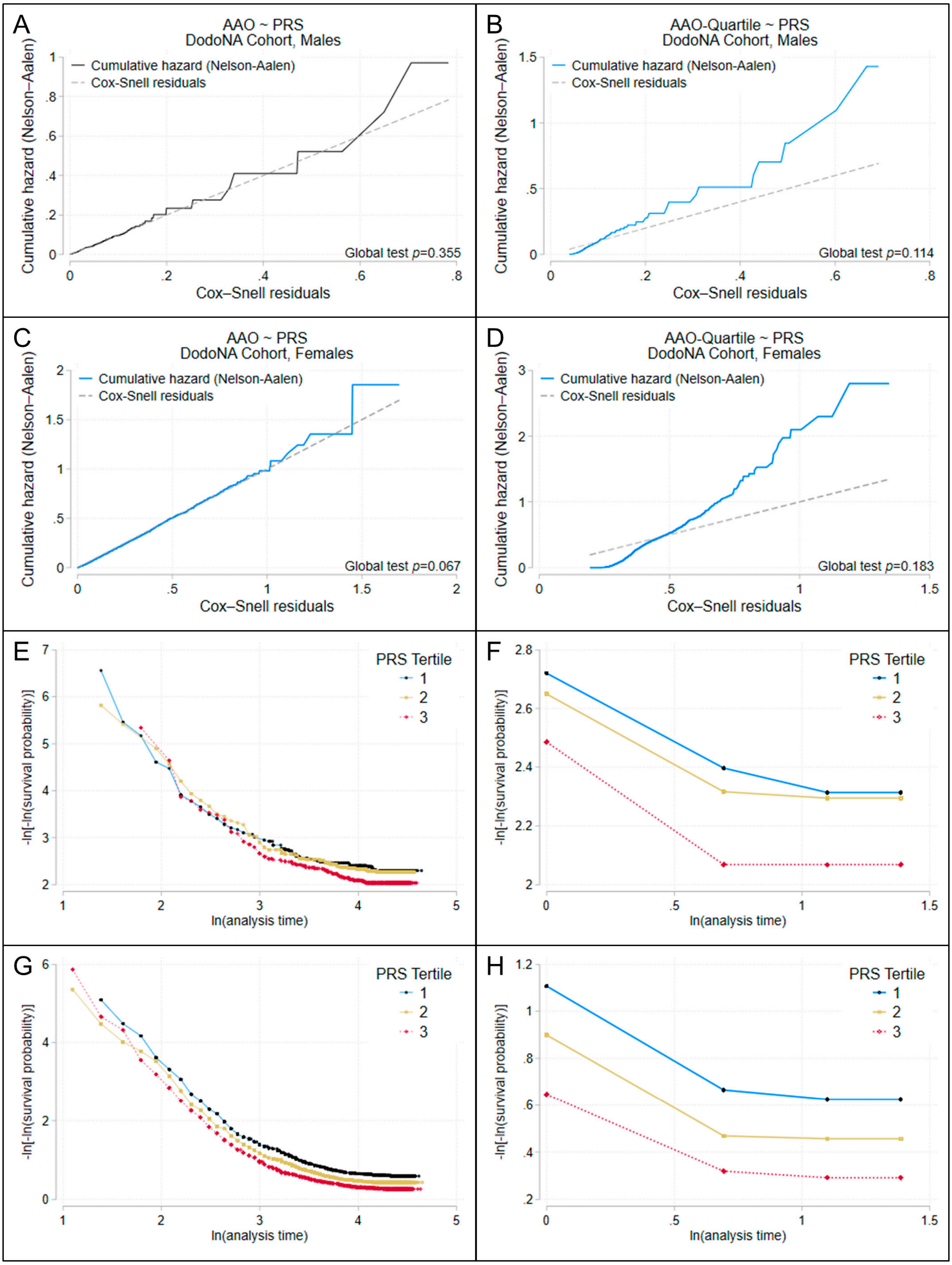

Supplemental Figure S1, part 2

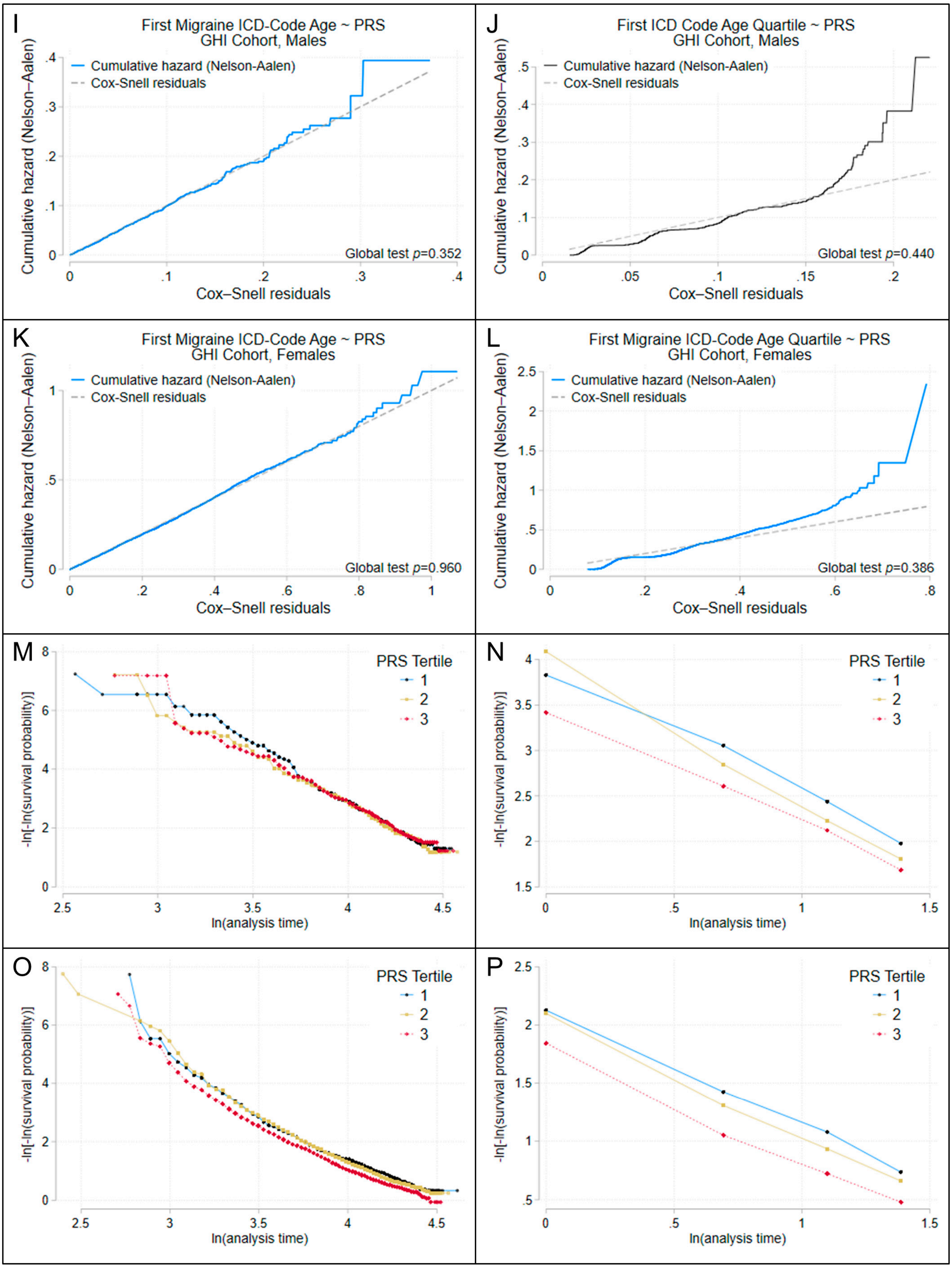

Supplemental Figure S1, part 3

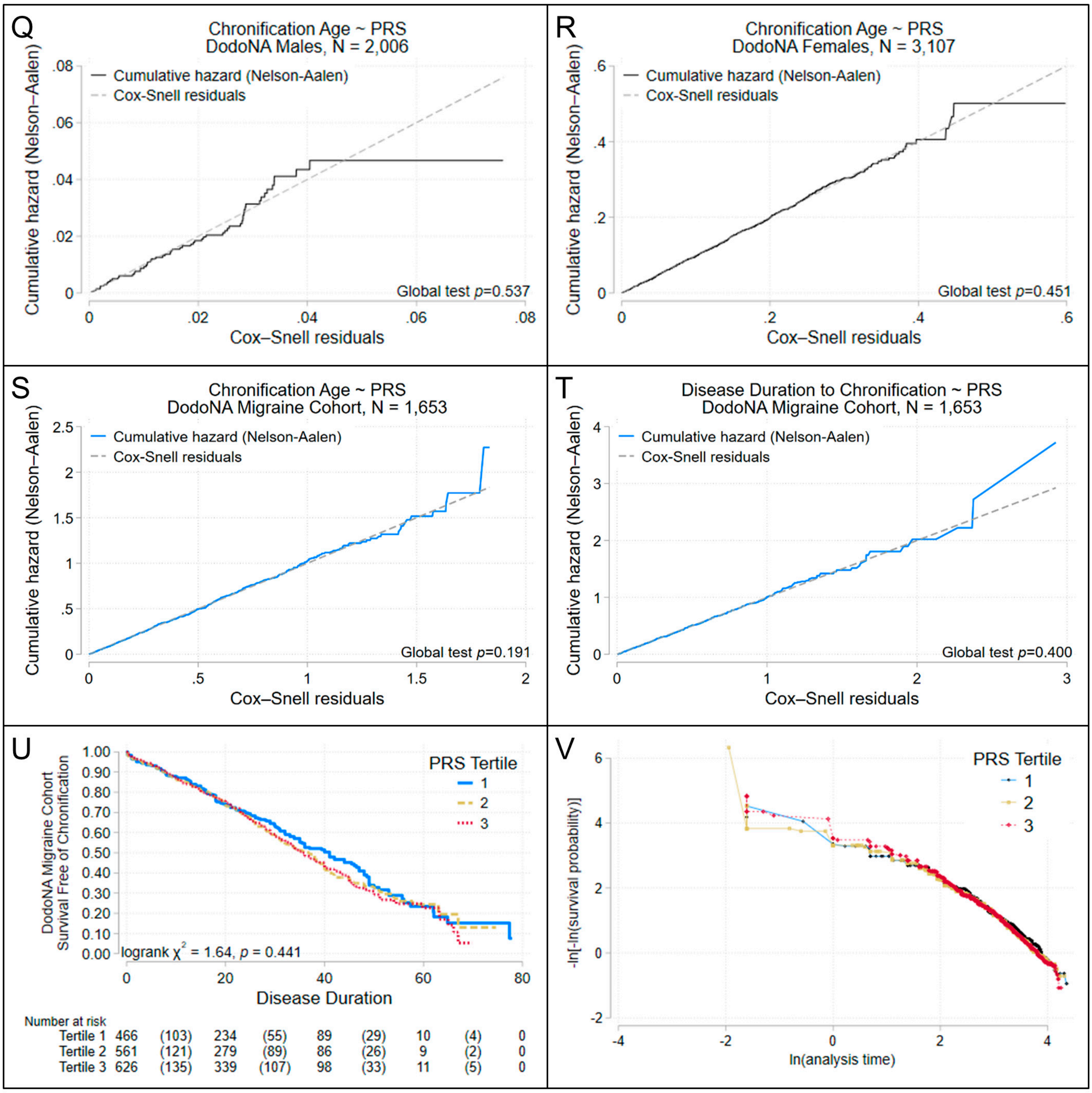

Supplement: Supplementary file 1 [file jcm-13-06483-s001.zip › jcm-3205459-supplementary.pdf]
